# Lack of Association between *TLR4* Genetic Polymorphisms and Diabetic Nephropathy in a Chinese Population

**DOI:** 10.1155/2014/704167

**Published:** 2014-03-23

**Authors:** Danfeng Peng, Jie Wang, Jiemin Pan, Rong Zhang, Shanshan Tang, Feng Jiang, Miao Chen, Jing Yan, Xue Sun, Tao Wang, Shiyun Wang, Yuqian Bao, Weiping Jia

**Affiliations:** Shanghai Diabetes Institute, Shanghai Key Laboratory of Diabetes Mellitus, Shanghai Clinical Center for Diabetes, Shanghai Key Clinic Center for Metabolic Diseases, Department of Endocrinology and Metabolism, Shanghai Jiao Tong University Affiliated Sixth People's Hospital, Shanghai 200233, China

## Abstract

*Objective*. Toll-like receptor 4 (TLR4) plays a central role in innate immunity. Activation of innate immune response and subsequent chronic low-grade inflammation are thought to be involved in the pathogenesis of diabetic nephropathy. In this study, we aimed to investigate whether *TLR4* variants are associated with diabetic nephropathy in the Chinese population. *Methods*. Seven tagging single nucleotide polymorphisms (SNPs) of *TLR4* based on HapMap Chinese data were genotyped in 1,455 Chinese type 2 diabetic patients. Of these patients, 622 were diagnosed with diabetic nephropathy and 833 were patients with diabetes for over 5 years but without diabetic nephropathy. *Results*. None of the SNPs and haplotypes showed significant association to diabetic nephropathy in our study. No association between the SNPs and quantitative traits was observed either. *Conclusion*. We concluded that common variants within *TLR4* genes were not associated with diabetic nephropathy in the Chinese type 2 diabetes patients.

## 1. Introduction

Type 2 diabetes has become an epidemic all around the world, resulted in large loss in economy, and threatened the human health. Diabetic microvascular complications are the major causes of morbidity and early mortality in diabetes [1, 2]. As one of the most important long-term complications of diabetes, diabetic nephropathy is the leading cause of chronic kidney failure and end-stage renal disease [3], and patients with diabetic nephropathy have an increased risk of cardiovascular mortality [4, 5]. At present, it is widely accepted that diabetic nephropathy is a heterogeneous disorder caused by the interaction between environmental factors and genetic factors. Diabetes duration and glycemic control are the strongest environmental risk factors [6], and family history of kidney disease appears to be the strongest risk factor for initiation of diabetic nephropathy [7, 8]. Recently, several studies have suggested that the innate immunity changes may be associated with type 2 diabetes and diabetic complications [9–11]. Thus, genes encoding the innate immune system components might be good candidates for studying diabetic nephropathy.

Toll-like receptors (TLRs) are the family of type I transmembrane receptors involved in innate immunity and pathogen recognition [12]. While toll-like receptor 4 (TLR4) is predominantly expressed on dendritic cells and macrophages and plays an important role in the activation of the innate immune response and subsequent proinflammatory reactions, it not only recognizes the lipopolysaccharide (LPS) of Gram-negative bacteria, but also interacts with some endogenous ligands, such as heat-shock proteins, fibronectin, oxidized low-density lipoprotein cholesterol, Fetuin-A, and high-mobility group box 1 (HMGB1) [12–17]. Several studies have indicated that there may be a link between TLR4 pathway and diabetic nephropathy [18–20]. Therefore, in this study, we aim to investigate whether* TLR4* genetic polymorphisms are associated with diabetic nephropathy and its related quantitative traits in the Chinese population.

## 2. Methods

### 2.1. Participants

This study involved 1,455 patients with type 2 diabetes recruited from the Shanghai Diabetes Institute Inpatient Database of Shanghai Jiao Tong University Affiliated Sixth People's Hospital. All participants were unrelated type 2 diabetic patients meeting the 1999 WHO criteria. Of these patients, 622 were diagnosed with diabetic nephropathy and 833 were patients with diabetes for over 5 years but without diabetic nephropathy, considered as cases and controls for diabetic nephropathy, respectively. This study was approved by the institutional review board of Shanghai Jiao Tong University Affiliated Sixth People's Hospital, with written informed consent obtained from each participant.

### 2.2. Clinical Measurement

The 24 h albumin excretion rates (AERs) and estimated glomerular filtration rate (eGFR) were applied to asses nephropathy. AERs were measured in 3 consecutive days, and the mean value was recorded for each patient. Patients with AER < 30 mg/24 h, 30 mg/24 h ≤ AER < 300 mg/24 h, or AER ≥ 300 mg/24 h were classified as having normoalbuminuria, microalbuminuria, or proteinuria, respectively. Patients having microalbuminuria or proteinuria were diagnosed with diabetic nephropathy. eGFR was calculated using a formula developed by the Modification of Diet in Renal Disease study group with adjustment for Chinese ethnicity: 186 × (serum  creatinine  in  mmol/L × 0.011)^−1.154^ × (age  in  years)^−0.203^ × (0.742 if female) × (1.233 if Chinese) [21]. Glycemic control was evaluated by measuring glycated HbA1c levels. Data of blood pressures and lipid profiles were also collected for each participant.

### 2.3. Single Nucleotide Polymorphisms (SNPs) Selection, Genotyping, and Quality Control

We selected tagging SNPs according to HapMap phase III (release 28) Han Chinese database with a threshold of *r* ^2^ > 0.8 by using Haploview (v 4.2). The seven tagging SNPs selected could cover 100% of common SNPs (14 out of 14 SNPs in the HapMap Chinese Han samples) with a minor allele frequency (MAF) > 0.05. All genotyping was done using the primer extension of multiplex products with detecting by matrix-assisted laser desorption ionization time of flight mass spectroscopy using a MassARRAYCompact Analyzer (Sequenom, San Diego, CA, USA). The genotyping data underwent a series of quality control checks as described previously [22] and cleared data were used in further statistical analysis. Overall, 2 individuals were excluded from the sample call rate checks and all the seven SNPs passed the SNP call rate check with an average call rate of 98%.

### 2.4. Statistical Analysis

The Hardy-Weinberg equilibrium test was performed before the association analysis (a two-tailed *P* value < 0.05 was considered statistically significant). The allelic frequencies between the patients with or without diabetic nephropathy were compared by *χ* ^2^ test, and odds ratios with 95% confidence intervals (CIs) were presented. Linear regression was applied to test the effect of genotype on quantitative traits with adjustment of confounding factors under an additive model. Skewly distributed quantitative traits (eGFR and AER) were logarithmically transformed (log 10) to approximate normality before linear regression analysis. All these analyses were performed using SAS 9.3 (SAS institute, Cary, NC, USA) unless specified otherwise. A two-tailed *P* value < 0.05 was considered statistically significant.

## 3. Results

**Figure 1:**
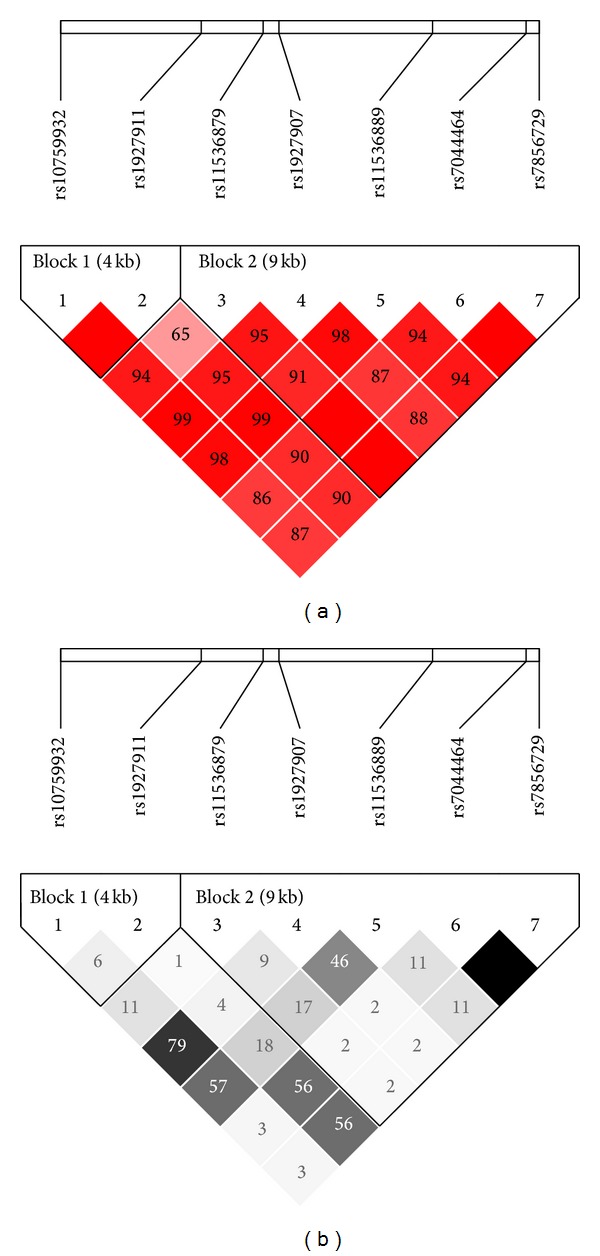
Linkage disequilibrium maps for SNPs genotyped in* TLR4* region. (a) Shades of red demonstrate the strength of the pairwise linkage disequilibrium based on D′ and numbers represent the value of D′ expressed as a percentage. (b) Shades of grey show the strength of the pairwise linkage disequilibrium based on *r* ^2^ and numbers indicate the value of *r* ^2^ expressed as a percentage.

All the seven SNPs were in accordance with Hardy-Weinberg equilibrium. The clinical characteristics of the samples passed genotype quality control were shown in Table 1. The linkage disequilibrium pattern of these SNPs was shown in Figure 1. Two haplotype blocks were constructed in this region.

The single SNP association analysis showed that no SNP was significantly associated with diabetic nephropathy in our samples. The minimum *P* value was 0.417 for rs7044464 (Table 2). Then we compared the frequencies of haplotypes between type 2 diabetic patients with or without diabetic nephropathy and observed that there was no nominal difference between two groups either (Table 3). Further, we analyzed the effect of these SNPs on nephropathy related quantitative traits. With adjustment of age, sex, body mass index, duration of diabetes, HbA1C, diastolic pressure, and systolic pressure, we found that no SNP was nominally associated with AER and eGFR. The minimum *P* value was 0.087 for the association between rs10759932 and AER (Table 4).

## 4. Discussion

It has been shown that TLR4 and its signal pathway participated in the pathogenesis of diabetes and diabetic nephropathy. Dasu et al. [23] reported that* TLR4* expression and its ligand, signaling, and functional activation were increased in recently diagnosed type 2 diabetes subjects and contributed to the proinflammatory state. Furthermore, knockout of* tlr4* attenuated the proinflammatory state of diabetes in animal models [24]. Exposure of isolated islets of Langerhans to LPS reduced insulin gene expression; insulin secretion was inhibited as well. However, those effects were not observed in islets from TLR4-deficient mice [25]. In vitro, TLR4 expression and activity were increased under hyperglycemia in mesangial cells and could contribute to the progression of diabetic nephropathy [18]. Liu et al. [20] observed that renal TLR4 expression was significantly higher in diabetic nephropathy in animal model, as well as kidney/body weight ratio, serum creatinine, CRP, and TNF-*α* level. TLR4 has also been proved to accelerate the progression of diabetic nephropathy induced by hyperlipidemia [26]. The research of Lin et al. [17] also showed that TLR4 pathway promoted tubular inflammation in diabetic nephropathy. And their further study investigated that TLR4 antagonist CRX-526 could reduce albuminuria and blood urea nitrogen without altering blood glucose and systolic blood pressure in diabetic mice, thus protecting diabetic mice from advanced nephropathy [19]. Above all, it is convincible that TLR4 and its signal pathway play an important role in diabetic nephropathy.

The data of* TLR4* genetic polymorphisms and diabetic complications was limited. It was reported in a German population that Asp299Gly and Thr399Ile genotypes of the* TLR4* were associated with diabetic neuropathy in type 2 diabetes, but not with diabetic nephropathy [27]. Asp299Gly was associated with early onset of diabetic retinopathy in the type 2 patients, reported in a Poland population [28]. However, these two polymorphisms were not detected in the Chinese population [29–32]. In the present study, we aimed to investigate the association between* TLR4* polymorphisms and diabetic nephropathy. However, we failed to find any evidence of association between SNPs from this locus and traits related to diabetic nephropathy in our samples. One possible explanation might be that the statistical power of our samples was not enough to detect the effects of this locus in the Chinese population. With our samples, we had over 75% power to detect an effect SNP (OR = 1.4) with minor allele frequency of 0.1 at a 0.05 level. We could not exclude the possibility that associated SNPs with lower minor allele frequency or lower effect in the Chinese existed. Secondly, although the tagging SNPs we selected covered all the common SNPs of* TLR4*, it is still possible that rare variants of* TLR4* associated with diabetic nephropathy existed. Thirdly, in our study, the diagnosis of diabetic nephropathy was not determined by the histological analysis of tissue samples obtained from renal biopsies, which is the golden standard [33]. So we cannot exclude the possibility that patients diagnosed with diabetic nephropathy also may include nondiabetic renal disease and a superimposed nondiabetic condition on underlying diabetic nephropathy. However, we excluded patients with history of renal diseases in the enrollment of study subjects, and the blood pressures of patients with or without diabetic nephropathy were similar, and thus the influence of the diagnostics of diabetic nephropathy on our study was limited.

## 5. Conclusions

In conclusion, although many functional researches have implied that TLR4 played an important role in diabetic nephropathy, our study suggested that common variants within* TLR4* gene were not associated with diabetic nephropathy in the Chinese type 2 diabetes patients. However, due to the limitation of the current study, the effects of SNPs from this locus on diabetic nephropathy needed to be tested in further studies with larger samples with accurate diagnosis for diabetic nephropathy.

## Figures and Tables

**Table 1:** Clinical characteristics of the study samples.

	Cases	Controls
Samples (*n*)	621	832
Male/female (*n*)	348/274	352/480
Age (years)	62.42 ± 12.67	63.31 ± 10.56
BMI (years)	24.95 ± 3.86	24.00 ± 3.35
Age at diagnosis of diabetes (years)	53.56 ± 12.27	42.38 ± 10.22
Duration of diabetes (years)	9.00 (3.00, 14.00)	10.00 (7.00, 14.00)
Hemoglobin A_1C_ (%)	9.34 ± 2.29	8.68 ± 2.10
Systolic blood pressure (mmHg)	140.04 ± 19.27	134.52 ± 18.27
Diastolic blood pressure (mmHg)	82.53 ± 9.90	79.57 ± 9.05
AERs (mg/24 H)	93.09 (47.47, 296.27)	9.23 (6.40, 14.58)
eGFR*	109.81 (81.62, 136.87)	121.57 (103.40, 144.84)

Data are *n*, mean ± SD, or median (interquartile range). BMI: body mass index. AERs: albumin excretion rates. eGFR: estimated glomerular filtration rate. eGFR* was calculated by using a formula developed by the Modification of Diet in Renal Disease study group with adjustment for Chinese ethnicity.

**Table 2:** Associations of *TLR4* SNPs with type 2 diabetic nephropathy.

SNP	Chr. position (Build 37.3)	Major/minor allele	Risk allele	Cases		Controls		OR for minor allele (95% CI)	*P* value
				(*n* = 621)		(*n* = 832)			
				Minor allele frequencies	Genotype count 11/12/22^#^	Minor allele frequencies	Genotype count 11/12/22^#^		
rs10759932	9:120465144	T/C	T	0.310	290/236/65	0.309	392/336/83	1.001 (0.851–1.177)	0.993
rs1927911	9:120470054	C/T	T	0.435	201/289/121	0.427	267/403/148	1.030 (0.887–1.196)	0.698
rs11536879	9:120472211	A/G	G	0.125	471/129/12	0.120	630/169/13	1.047 (0.835–1.312)	0.691
rs1927907	9:120472764	G/A	A	0.272	320/218/51	0.260	449/294/62	1.064 (0.894–1.260)	0.477
rs11536889	9:120478131	G/C	G	0.221	371/224/25	0.216	511/275/41	1.031 (0.862–1.231)	0.741
rs7044464	9:120481397	T/A	A	0.093	503/110/2	0.084	694/127/6	1.113 (0.859–1.443)	0.417
rs7856729	9:120481856	G/T	T	0.093	506/111/2	0.086	691/131/6	1.084 (0.838–1.402)	0.541

^#^11, major allele homozygotes; 12, heterozygotes; 22, minor allele homozygotes.

The OR with 95% CI shown is for the minor allele.

**Table 3:** Associations of two haplotypes in *TLR4* region with diabetic nephropathy.

Haplotype	Haplotype frequencies		*P* value
	Cases	Controls	
Block 1 (rs10759932-rs1927911)			
TA	0.566	0.570	0.827
CA	0.309	0.309	0.973
TG	0.125	0.120	0.705
Block 2 (rs11536879-rs1927907-rs11536889-rs7044464-rs7856729)			
GGCTG	0.342	0.361	0.288
GATTG	0.269	0.253	0.341
CGCTG	0.215	0.205	0.520
GGTAT	0.088	0.080	0.404
GGTTG	0.071	0.081	0.320

**Table 4:** Associations of *TLR4 *SNPs with clinical features related to diabetic nephropathy in type 2 diabetic patients without nephropathy.

SNP	AERs			eGFR		
	Beta	SE	*P* value^#^	Beta	SE	*P* value^#^
rs10759932	−0.022	0.013	0.087	0.003	0.006	0.683
rs1927911	−0.011	0.012	0.354	0.002	0.006	0.781
rs11536879	0.019	0.019	0.312	−0.001	0.009	0.894
rs1927907	−0.019	0.013	0.165	0.001	0.006	0.839
rs11536889	0.000	0.015	0.974	−0.003	0.007	0.712
rs7044464	0.022	0.022	0.314	0.002	0.011	0.865
rs7856729	0.024	0.022	0.271	0.006	0.011	0.581

The additive model was used in the analysis.

*P* value^#^ was adjusted for age, sex, BMI, diabetes duration, HbA1C, and blood pressure.
